# Address

**Published:** 1873-12

**Authors:** J. C. Ross

**Affiliations:** President of the Tennessee Dental Association


### ADDRESS.

BY J. C. ROSS, D. D. S., PRESIDENT OF THE TENNESSEE DENTAL ASSOCIATION.

Gentlemen of the Tennessee Dental Association :—On retiring from the chair as your presiding officer, allow me to congratulate you on the peace and harmony which have characterized our meeting, and to thank you for the courtesy and kindness extended to me personally, while presiding over your deliberations.

The Tennessee Dental Association was organized in July, 1867. Though we have not perhaps exerted ourselves to the extent we should, nor have we increased in numbers and influence as rapidly as we might have done, still the Society has not been without its beneficial results and its influence for good —especially in Middle Tennessee.

In a recent number of the DENTAL REGISTER is a well-written article entitled "The Dentist of the Future." The author, in entering upon the subject, throws a glance backward on the early history of dentistry in this country, and pays a just tribute to the memory of Drs. Harris, Parmely, Badger and others who were pioneers in the profession, and who did so much to inaugurate the higher tone and the more liberal sentiments which now pervade and actuate the members of the profession throughout the length and breadth of this country.

The progress of dental science is the constant theme of those who write for our dental journals ; and it is certainly a matter of just pride to those engaged in our beloved and time-honored profession, that it is advancing so rapidly.

Prof. T. O. Summers, though not of the dental profession, in a published letter, not long since, referring to the Southern Dental Association, recently convened at Baltimore, says ; "I find among them (the dentists) such a wonderful spirit of advancement and progress that it always stimulates me to larger efforts and more earnest investigation."

Such an expression of commendation from a gentleman of Prof. Summers' scientific attainments and opportunities for observation should incite us to more zeal and energy in the investigation of truth.

Young men who are now entering the dental profession have probably but little conception of the many and superior advantages which they possess over those of us who began the practice thirty-five years ago. There was not then a dental college in America. There were but few—very few—standard works, and they of foreign authorship, and there was not a single dental periodical. In a word, there was scarcely any American dental literature.

There were at that time no national or state associations, and few, if any, local societies.

The dental student who was seeking light and thirsting for knowledge was made to feel that the means for obtaining it were indeed meager.

There was not then the kindly and generous disposition manifest by the older members of the profession towards the younger, that we now see indicated. With a few honorable exceptions, it was every man for himself and every man against every other man in the profession.

How changed the aspect and condition of affairs now. Instead of a feeling of antagonism and rivalry, as heretofore, it is now one of affinity and kindly regard, when dentists meet.

We have now numerous standard works, and by our own American authors, men of first-class ability and scientific research ; among them, a Dictionary of Dental Science, by that great luminary in the galaxy of worthy contributors to dental literature, Prof. C. A. Harris.

We have also nine dental colleges, at either of which the student may in a few months acquire more information than heretofore he could obtain in as many years. Besides these, we have several excellent periodicals devoted exclusively to the interests of the profession, which contain much valuable information in dental science, and serve as an advertising medium of the various and multiform inventions and appliances which are being introduced to facilitate and perfect our operations.

But, probably, there is no one thing which has contributed more largely to the improvement and advancement in the art and science of dentistry, than that of association. It is by associated effort that great and important results are produced in almost any enterprise. By free discussion and unreserved inter-communication, and comparing observation and experience with observation and experience, we can learn, one from another, that which cannot be obtained from books and other sources.

In our convocations, whether in the capacity of local societies, or as district, state or national organizations, it should be an object ever kept in view, that we meet for mutual improvement, and that we come with something to contribute to the general fund of knowledge as well as to learn—something to give as well as to receive. This is the spirit and these the sentiments that should ever actuate and influence those engaged in the same profession.

